# Dynamic secondary electron emission in rough composite materials

**DOI:** 10.1038/s41598-019-50353-3

**Published:** 2019-09-27

**Authors:** Leandro Olano, Maria E. Dávila, John R. Dennison, Petronilo Martín-Iglesias, Isabel Montero

**Affiliations:** 1 Instituto de Ciencia de Materiales de Madrid, CSIC, C/Sor Juana Inés de la Cruz 3, 28049 Madrid, Spain; 2 Utah State University Dept. of Physics, 4415 Old Main Hill, Logan, UT 84322-4415 USA; 3 European Space Agency, ESA. Keplerlaan 1, 2201 AZ Noordwijk, The Netherlands

**Keywords:** Materials science, Materials for devices

## Abstract

The interaction of ionizing radiation with matter is of critical importance in numerous areas of science and technology like space and vacuum technology and even medicine and biotechnology. Secondary electron emission is a consequence of electron irradiation on materials. We achieve extremely low secondary electron emission yield values smaller than 0.2, even up to incident electron energies ~1 keV, due to an undocumented synergy between neighbouring metal and dielectric domains in composite samples. To investigate this experimental discovery, we propose a simple 3D model where the dielectric and metallic domains are arranged in parallel and interleaved. The proposed surface profile has a triangular shape to model the surface roughness. We obtain a continuous equation to describe the electric field that arises between grounded conductors and charged dielectrics domains. The calculated trajectories of secondary electrons in this 3D geometry are used to predict dynamic secondary emission yield, which strongly depends on the charge accumulated in the dielectric domains. This research paves the way to design new materials of low secondary emission yield, addressing the technological problem not yet resolved to inhibit the electron avalanche in RF equipment that limit their maximum working power.

## Introduction

The effects of exposure to ionizing radiation is of great importance in different areas of science and technology from space and vacuum technology to even medicine and biotechnology. The effect of electron irradiation on matter is the ionization of the atoms in the material. The excited electrons travel through the material until they either lose their energy and are reabsorbed or arrive at the surface and are emitted as secondary electrons, SE.

Secondary electron Emission Yield (SEY) characterizes the number of electrons emitted by a material when an electron irradiation (primary electrons) impinges on its surface. SEY, usually denoted by *σ*, is defined as the ratio between the total number of emitted electrons and the total number of incident electrons. As well as being dependent on the material, SEY is a function of the energy (primary energy) and angle of incidence of the primary electron (PE) beam. SEY curves are generally plotted in terms of the PE energy for an electron beam normal to the surface. The shape of SEY curves, which mainly depends on the surface. The cross-over energies are defined as the energies below and above E_max_ for which *σ* = 1.

SEY is a limiting factor for many vacuum-related industries and therefore has a great economic importance. For example, a high SEY is the main cause of the onset of an electron avalanche, called multipactor effect, in high- power RF devices in space^1–3^, as well as the electron cloud (EC) effect in large accelerators^4–6^. It is also fundamental for other charging phenomena in satellites^7^. The multipactor effect develops when free electrons are accelerated by the electric field of an RF signal transmitted through an RF device, hitting its inner walls and consequently emitting secondary electrons. When these secondary electrons enter into resonance with the RF signal, they repeatedly hit the inner walls, increasing steadily the population of electrons if *σ* > 1. This process continues as long as the signal is sustained, until an unavoidable electron avalanche occurs. This electron avalanche induce malfunctions and permanently damage RF devices. In the case of a satellite in space, it can even cause the failure of the mission.

The resonance conditions of the multipactor discharge can often be inhibited by an adequate design of parameters pertaining to the RF electromagnetic field, but there always remain some critical regions where resonance conditions can only be avoided by using surfaces with low secondary electron emission. For this reason, one of the main efforts for multipactor inhibition is based on reducing the SEY of surfaces prone to multipactor discharge^5,8–11^. As the emission of secondary electrons is a surface process, only the exposed surface of the material needs to be modified. Conceptually, the most suitable materials for space applications are those with *σ*_max_ < 1. In this case, the number of secondary electrons emitted is less than the number of primary electrons hitting the surface, for all primary electron energies, so that the electron population decreases over time and the electron avalanche is prevented.

A more complex behaviour appears when the material exposed to electron irradiation is dielectric. Secondary electron emission causes charge to build up on vacuum-exposed dielectric surfaces. Additionally, high-energy PEs can penetrate the dielectric material, leading to the development of thicker charged regions. The interaction between the primary or secondary electrons with the electric field generated by the deposited charge makes the SEY of dielectric materials tend to 1 as the accumulated charge increases. Eventually, the inbound and outbound charge fluxes compensate each other, stabilizing the total deposited charge. For example, for *σ* > 1 (i.e., primary energies between E_1_ and E_2_), some of the emitted SEs are re-attracted by the electric field. This effect decreases the number of emitted electrons, decreasing the effective SEY and leading to *σ* ≅ 1 at the equilibrium surface potential. For *σ* < 1 (i.e., primary energies lower than E_1_ or higher than E_2_), the primary electrons are slowed down by the electric field due to accumulated charge until their incident energy yields *σ* ≅ 1 or until they are repelled from the surface^12–14^. The first case is called positive charging regime, while the second case is called negative charging regime. These effects can cause problems with dielectric materials in vacuum exposed to electron irradiation. The voltage gradients created can be large, and discharges between charged and grounded components can have serious consequences^15–17^.

Composite materials with tuneable properties are used in a wide range of industries^18,19^. SEY measurements for some smooth composites have been reported in the literature^20^, but none of these works describe an interaction between metals and dielectric domains to decrease the electron emission. In this work we present an undocumented synergy between the charging capacity of dielectric domains and the conductivity of conductor domains, which decreases the SEY of the coating and stabilizes SEY at lower values. In essence, the electric field that arises between grounded conductors and charged dielectrics decreases the SEY, by driving SEs back to the conducting sample. In this way, it is possible to achieve extremely low SEYs even for high primary energies^21^, which is highly desirable for a wide range of technological applications. Other studies in the literature report low SEY of high aspect ratio surfaces^9–11,22–24^. In the present study we find even lower SEY values for much lower aspect ratios due to the electric field between the metal and dielectric domains of the coating.

In this paper, we describe experimental measurements of SEY curves for three rough composite materials with very different compositions and properties, chosen for their relevance to space RF devices. Furthermore, we propose a theoretical model of the composite surface that successfully describes the observed experimental SEY behaviour and explains how low SEYs can be achieved in practical applications.

## SEY Measurement Procedure

In this work the SEY measurements of the samples were performed using two different methods. (i) In the continuous method, we irradiate the sample continuously with an electron beam and increase the energy of the PEs linearly with time. The total dose delivered to the surface were 10 nC/mm^2^ and 100 nC/mm^2^. (ii) In the pulse method, the primary beam is pulsed into 170 ns pulses, with each pulse delivering ~1 fC/mm^2^. In this case the energy also increases with time, and a single pulse is generated for each primary energy. SEY measurements were taken in an ultra-high vacuum (UHV) chamber with pressure <10^−9^ mbar. The electron sources were a Kimball Physics e-guns, delivering PE energies in the range 0 to 5000 eV.

In order to obtain the SEY of a sample the electron gun current, *Q*_*eGun*_, is previously calibrated. Then, the sample is irradiated and the current of the sample to ground, *Q*_*Ground*_, is measured by Keithley electrometers (continuous method) or a fast Femto amplifier and a Keysight oscilloscope (pulsed method). SEY is thus computed as follows

1 $$\sigma ={{Q}}_{{emit}}/{{Q}}_{{eGun}}=1-{{Q}}_{{Ground}}/{{Q}}_{{eGun}}$$

## Sample Description

We prepared three different types of rough composites with well-differentiated conductor and dielectric domains on the surface: (i) a dielectric epoxy resin mixed with Fe particles, which confer conductive properties on the resin (*Sample 1*); (ii) zeolites (NaAlSiO_6_-H_2_0) coated with gold nanoparticles (*Sample 2*); and (iii) a mixture of dielectric polyimide thermosetting resin and aluminium particles (*Sample* 3)^21^.

*Sample 1* was made from a powder of epoxy mixed with Fe particles. The mixture was moulded into solid cylinders of 30 mm diameter, then sliced into samples 2 mm thick. For *Sample 2*, zeolite particles were deposited on an aluminium substrate that was totally covered by adhesive conductive graphite tape. Gold nanoparticles were then deposited using a standard sputtering method, resulting in partial coverage by a gold layer ~2 nm thick. The same procedure was used to fix conductor and dielectric particles, with a size of 1 mm, in *Sample 3*.

**Figure 1:**
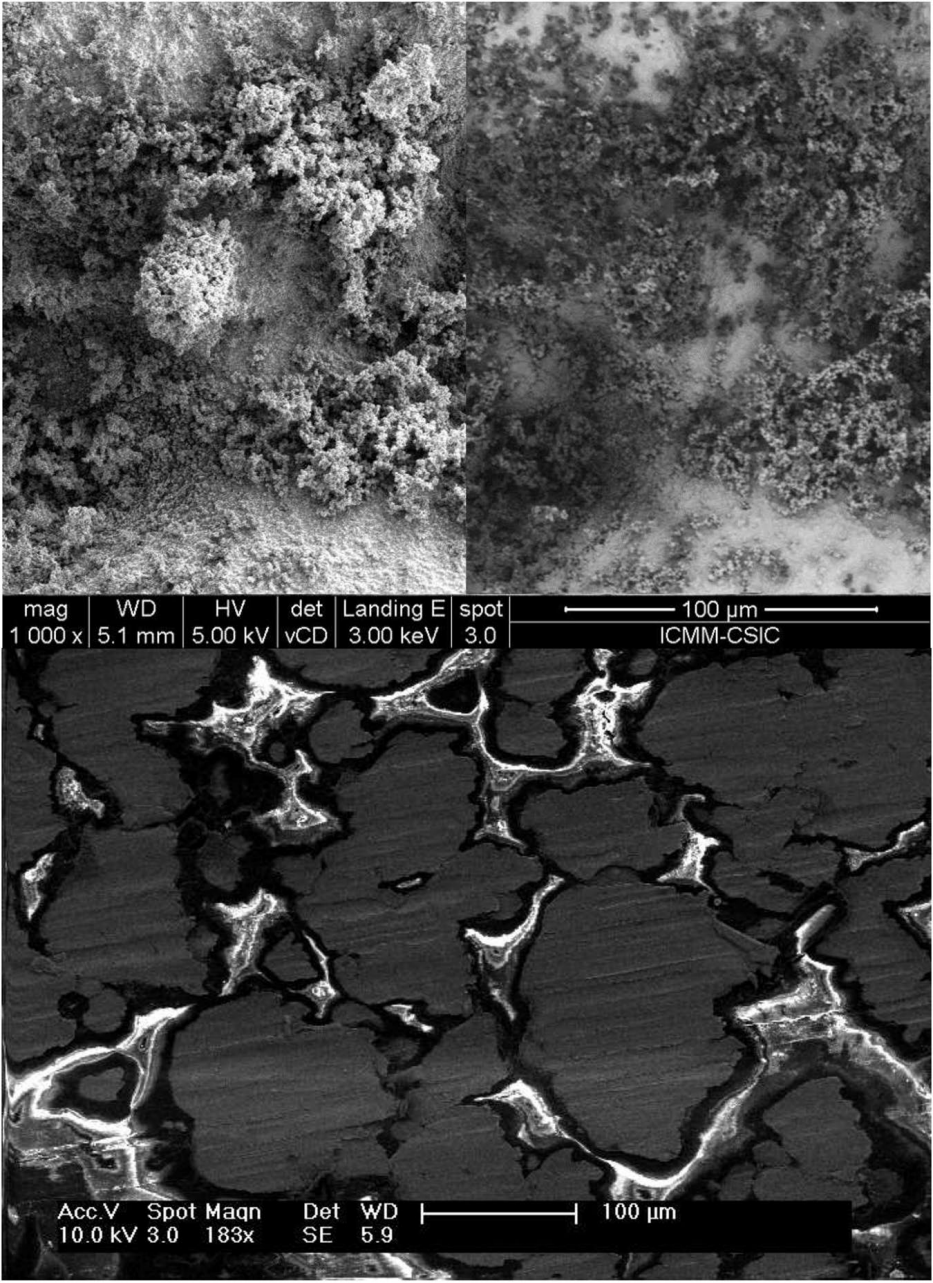
SEM images of Sample 1 and Sample 2. Top panel: SEM images of Sample 2. In backscattered mode (right, the bright areas are gold-coated domains, high Z, and the dark areas are uncoated zeolite regions. In secondary mode (left), the bright areas denote charging or high regions. Bottom panel: SEM image (secondary mode) of Sample 1. The bright areas are epoxy resin regions charging under the electron irradiation, while the dark areas are conducting Fe regions.

The surface morphology of the samples was analysed with a scanning electron microscope (SEM), as shown in Fig. 1. The SEM is able to qualitatively determine roughness, accumulated charge, and differences in composition. When the SEM is used in backscattering mode, bright areas represent elements with a bigger atomic number, Z, because the number of backscattered electrons increases with Z. This effect can be observed in the right image of the top panel of Fig. 1; the bright areas are the gold-coated domains of *Sample 2* and the dark areas are uncoated zeolite regions. The composition was also obtained by Energy Dispersive X-Ray spectroscopy, EDX. When SEM is used in secondary electron mode, surface morphology is presented in grey scale, but bright areas can also be due to charge accumulating in the sample. In the bottom panel of Fig. 1, charging is the main factor producing bright areas. This operating mode lets us differentiate the conducting and dielectric domains in the surface of *Sample 1*.

## SEY Results

**Figure 2:**
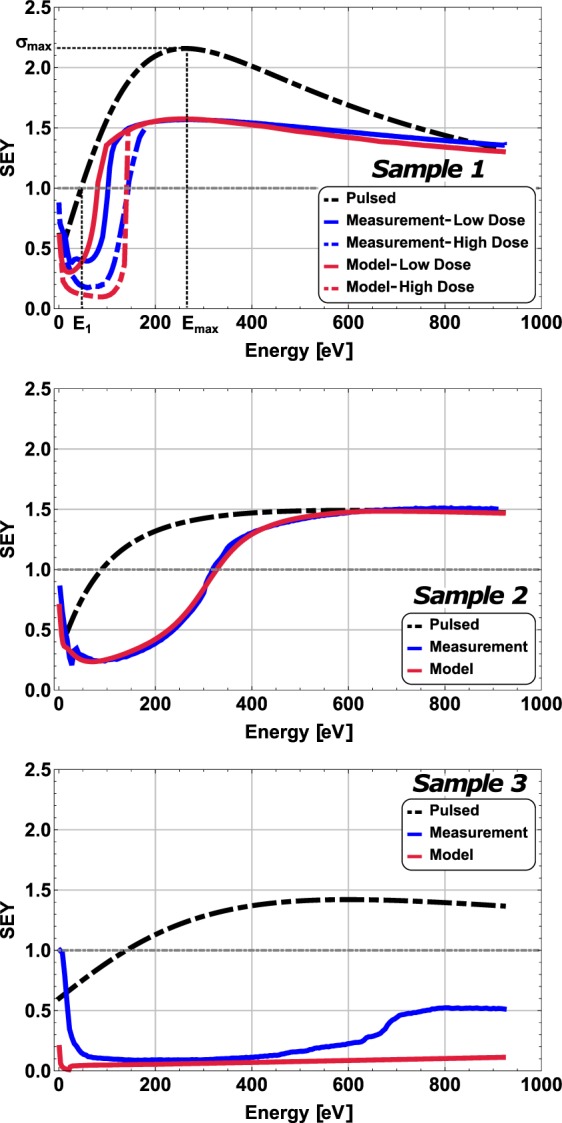
SEY measurements and model predictions. SEY as a function of the primary electron energy for *Samples* 1, *2*, and *3*. The measured SEY is given in blue, the SEY given by the model is in red, and the measured uncharged SEY is given in black. In the top figure the SEY parameters, E_1_, E_max_ and σ_max_, are shown.

Secondary emission yield measurements under electron bombardment of *Samples 1*, *2* and *3*, obtained by the continuous and pulsed methods, are shown in Fig. 2 and Table 1. There is a noticeable difference in the values of the first cross-over energy, energy at which *σ* = 1, measured by the pulsed and continuous methods. We name these parameters E_1_ and E_1_^C^ respectively, where the C stands for “continuous”. As we can observe in Fig. 2, E_1_^C^ > E_1_ for these samples. Specifically, for *Sample 1*, E_1_^C^ increases with the dose. It is especially remarkable that SEY values lower than 1 and close to 0.2 were measured in all samples, and even for primary energies up to about 1 keV in *Sample 3*.

**Table 1:** Dose, E_1_, *σ*_max_ and E_max_ parameters of the samples and its constituents.

	Dose (nC/mm^2^)	*E*_1_*/E*_1_^*C*^ (eV)	*E*_1_^*model*^*/E*_*1*_^*C*,*model*^ (eV)	*σ*_*max*_	*σ*_*max*_^*model*^	*E*_*max*_ (eV)	*E*_*max*_^*model*^ (eV)
*Sample 1*	1·10^−6^ (pulsed)	40.3	40.3	2.16	2.16	263.5	263.5
	10	101.9	80.0	1.57	1.57	276.2	256.0
	100	144.0	141.3	1.51	1.57	276.2	259.0
*Sample 2*	1·10^−6^ (pulsed)	88.6	88.6	1.49	1.49	601.0	601.0
	10	318.7	322.9	1.51	1.48	871.4	666.0
*Sample 3*	1·10^−6^ (pulsed)	140.0	140.0	1.42	1.42	601.0	601.0
	10	(a)	(a)	0.52	0.11	803.1	918.5
	*Constituents (b)*	***E***_***1***_ **(eV)**		***σ***_***max***_		***E***_***max***_ **(eV)**	
*Sample 1*	*Fe*	17.8		3.00		260.0	
	*Epoxy*	30.1		3.05		263.6	
*Sample 2*	*Au*	20.4		2.50		425.0	
	*Zeolite*	132.0		1.70		702.8	
*Sample 3*	*Al*	27.0		2.50		593.0	
	*TMM*	55.6		1.49		649.1	

The pulsed measurements are given to the model as inputs; therefore, the parameters of the pulsed measurements are the same as those used in the model.

(a) *σ* < 1 for all primary energies.

(b) SEY parameters of the constituents of composites for primary electron incidence at 45° and after exposure to air^26,29^.

Several charge relaxation mechanisms can take place in a dielectric sample, such as hopping charge transport, space-charge-limited conduction, and ohmic conduction^25^. The discharging process can be measured by delivering enough charge to the sample and controlling the SEY as it discharges towards the uncharged value. The resulting SEY decay curve is well fitted by a single exponential function with a time constant for charge release, *τ* ≈ 100 s.

## Dynamic SEY Model

To describe the SEY behaviour of the rough metal/dielectric coatings, we present here a unique model where the conductor and dielectric domains have the shape of infinitely long triangular prisms. The dielectric and conductor domains are arranged alternatively and in parallel on the sample plane of the sample substrate. This way the coating has a triangular profile that simulates the roughness of the samples. The model allows to easily change the roughness by using different prism shapes to represent the different domains. We chose an aspect ratio of 0.5 as a simple way of representing the general quality of surface roughness, and to avoid complexity.

The electric field is computed assuming that charge only accumulates on the surfaces of the dielectric domains. The image charge method is used to account for the grounded conductor domains. For simplicity, we use a nearest-neighbour approximation, where only the single conductor domain nearest to a given dielectric domain contributes to the mirror image charge. Under these assumptions, the source distribution is approximated as a set of infinitely long, uniformly charged strips placed on the external surfaces of the dielectric domains and their corresponding image charges. ${\vec{E}}_{Strip}\,(\vec{r})\,$is the electric field of a horizontal infinite strip, Eq. (2), with surface charge density Ω_Diel_ and unit width, with its center located at the origin of the coordinate system. Equation (3) represents the electric field, $\vec{E}(\vec{r})$, obtained by adding several infinite strips, with their directions and positions specified by the rotation and translation transformations ${ {\mathcal R} }_{\theta }$ and ${{\mathscr{T}}}_{{r}_{Diel/Image}^{0}}$ corresponding to the orientations and positions of dielectric and image strips, *r*^0^_*Diel*_ and *r*^0^_*Image*_. Five pairs of conductor and dielectric consecutive domains were used in the computation.

2 $${\vec{E}}_{Strip}\,(\vec{r})=\frac{{\Omega }_{Diel}}{4\pi {\varepsilon }_{0}}[Log(1+\frac{8x}{{(1+2x)}^{2}+4{z}^{2}}),\,\,0,2(ArcCot(\frac{2z}{1-2x})+ArcCot(\frac{2z}{1+2x}))]$$

3 $$\vec{E}(\vec{r})=\sum _{\{{r}_{Diel}^{0},\,\theta \}}{{\mathscr{T}}}_{{r}_{Diel}^{0}}\cdot { {\mathcal R} }_{\theta }\cdot {\vec{E}}_{Strip}(\vec{r}\text{'})-\sum _{\{{r}_{Image}^{0},\theta \}}{{\mathscr{T}}}_{{r}_{Image}^{0}}\cdot { {\mathcal R} }_{\theta }\cdot {\vec{E}}_{Strip}(\vec{r}\text{'})$$

**Figure 3:**
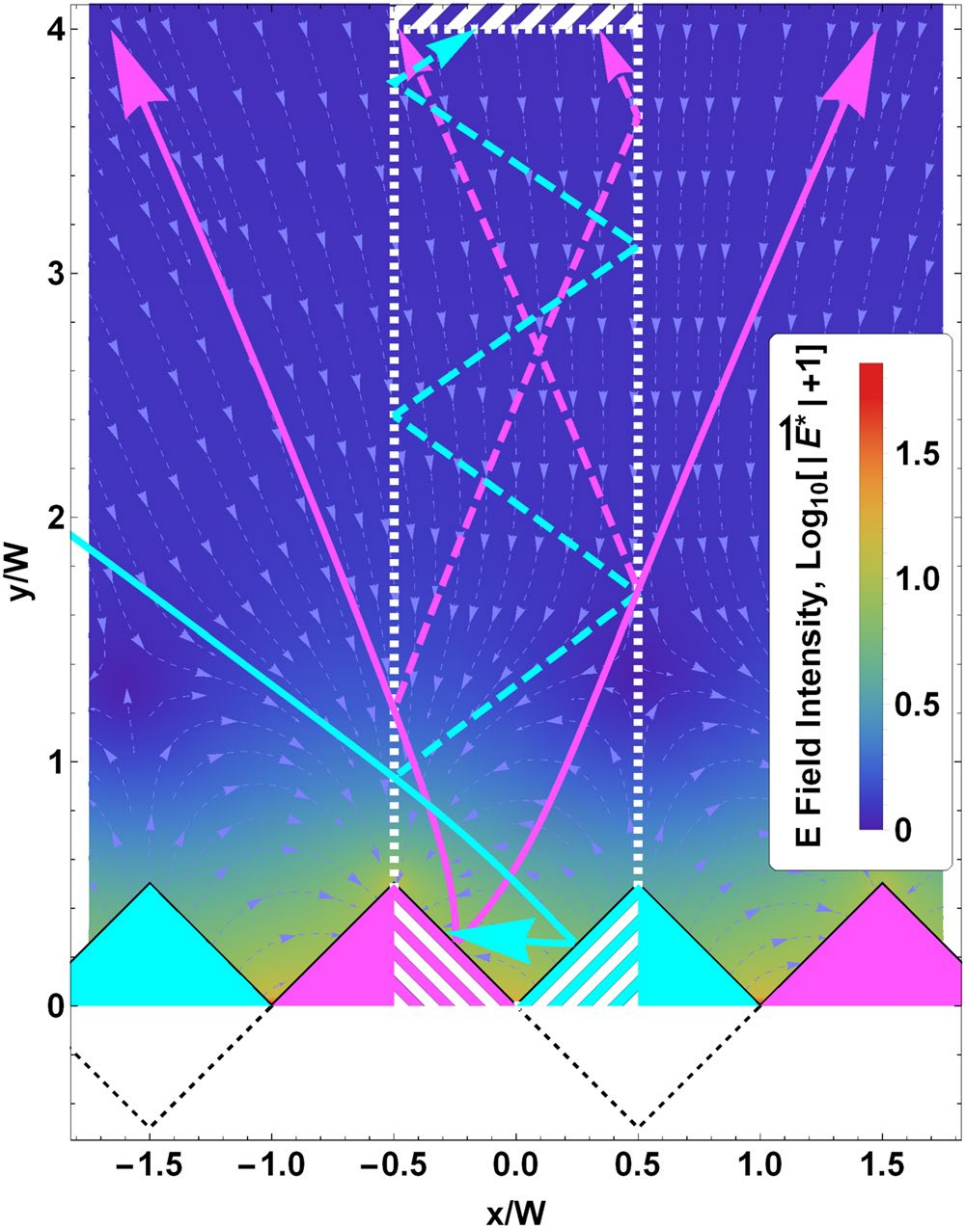
Transversal cross-section of the coating and the external electric field. The spatial dimensions are normalized to the prism width, W (see text). Magenta (cyan) triangles represent dielectric (conductor) domains. The positions of the image charges are shown as dashed black lines below the conductor domains. The vertical dashed lines represent the boundaries of the central region where boundary conditions are applied, and the white striped area at the top is where a SE is considered eligible for emission if its velocity is high enough. The light blue streamlines in the background represent the electric field direction but not its intensity. The background colours represent the dimensionless electric field $|\overline{{\boldsymbol{E}}{\boldsymbol{^{\prime} }}}|\equiv 4{\boldsymbol{\pi }}{{\boldsymbol{\varepsilon }}}_{0}/{{\boldsymbol{\Omega }}}_{{\boldsymbol{Diel}}}\cdot |\bar{{\boldsymbol{E}}}|$ in logarithmic scale. Magenta (cyan) coloured arrows represent SE trajectories generated in dielectric (conductor) domains as computed by the model.

As the charged strips have infinite length, the electric field is independent of the depth coordinate and the problem simplifies to two dimensions (see Fig. 3). However, simulating the SEY in 2D ignores any secondary electrons leaving the surface with a velocity component parallel to the prism axis. The electric field will have a bigger influence on such electrons, since they stay close to the surface longer. Thus, we expect the 2D simulation to underestimate the influence of surface charge on SEY. To avoid edge effects due to using a finite number of domains in the simulation, we apply periodic boundary conditions around the valley defined by the central pair of domains. This central area is delimited by the vertical dashed lines in Fig. 3. The figure length scale is normalized to the prism width, W. During the simulation, once an outbound SE rises to a distance where the electric field is normal to the surface (the dashed area at the top of Fig. 3), its velocity is compared to the escape velocity, calculated as the velocity at which the electron would escape considering the acceleration at its position. If this velocity is higher, the electron is considered to have been emitted by the sample. Otherwise, the computation continues until the SE fulfils the escape condition or hits a dielectric or conductor surface. In principle, the latter case could produce more SEs. However, such second-generation or tertiary SEs are unlikely for low incident energy SEs and are neglected in the model. This is further discussed below.

It is useful to state explicitly that the electric potential of this geometry is invariant if the surface charge density is inversely proportional to the domain size. This relationship is made explicit in Eq. (4), where $\mathop{r^{\prime} }\limits^{\longrightarrow}\equiv \vec{r}/W$ and $\mathop{E^{\prime} }\limits^{\longrightarrow}\equiv \vec{E}\cdot (4\pi {\varepsilon }_{0}/{\Omega }_{Diel})$ are the dimensionless spatial position and electric field.

4 $$\Delta V=-\,\int \vec{E}\cdot d\vec{r}=-\,\frac{W\cdot {\Omega }_{Diel}}{4\pi {\varepsilon }_{0}}{\int }^{}\overrightarrow{E^{\prime} }\cdot d\overrightarrow{r^{\prime} }$$

**Figure 4:**
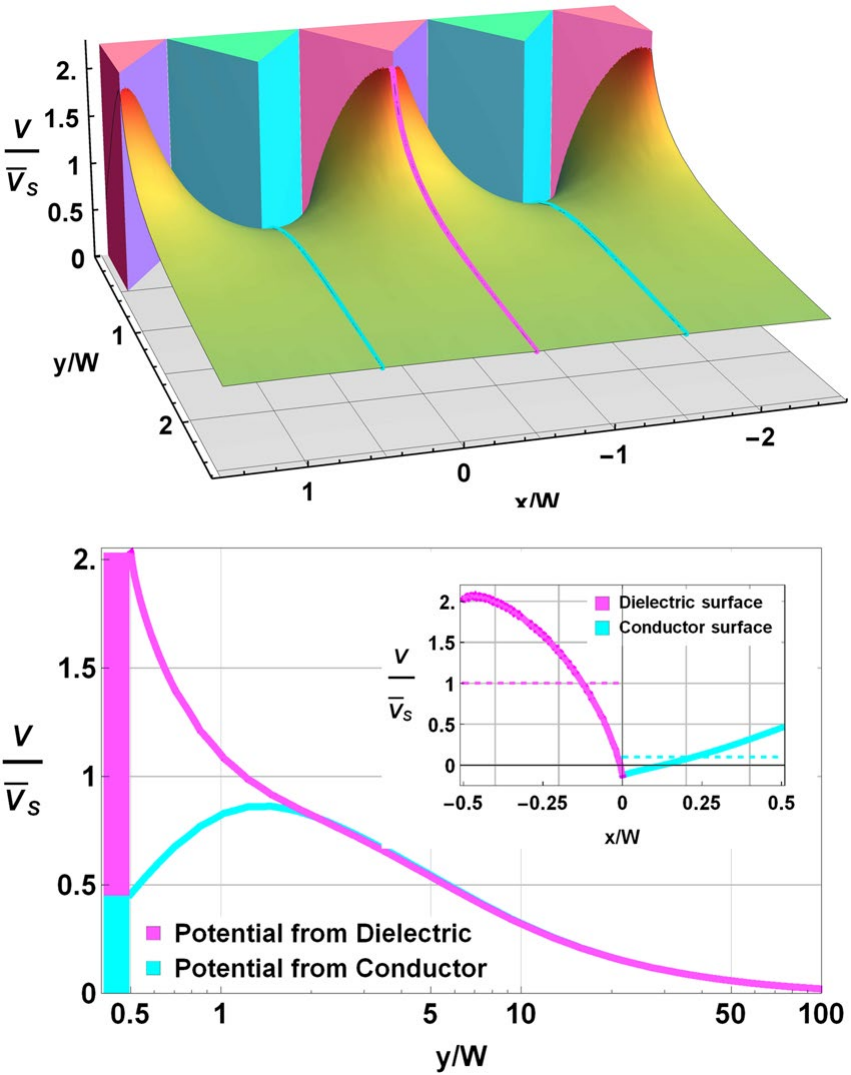
Potential well produced by a surface charge density in the metal/dielectric composite. The spatial dimensions are normalized to the prism width, W. Magenta (cyan) triangles represent dielectric (conductor) domains. (Top panel) Normalized external potential. A potential well appears over the conductor (dielectric) domains if the sign of $\overline{{{\boldsymbol{V}}}_{{\boldsymbol{S}}}}$ is negative (positive). (Bottom panel) Profile of the normalized potential from the top edge of the dielectric and conductor domains, as a function of the normalized distance over the sample. The profile shape corresponds to the magenta and cyan coloured gridlines in the top panel. Note that the voltage vanishes quickly. Inset shows the normalized voltage profile along the surface of the domains (solid line). Dashed lines are the mean surface voltages for the dielectric and conductor surfaces.

As the potential only depends on W and Ω_Diel_ once the geometry is set, we can compute a general potential for the system. This potential is graphed in Fig. 4, where we normalize it by $\overline{{V}_{S}}$ (mean voltage along the dielectric surface). Note that the shape of the external electric potential does not depend on the magnitude and sign of $\overline{{V}_{S}}$. Note also that, if $\overline{{V}_{S}} < 0$, the values of V are also negative and the shape of the non-normalized function $V(x/W,y/W)$ is reflected around the horizontal plane ($V/\overline{{V}_{S}}=0$) compared to the normalized function shown in Fig. 4.

A periodic potential well for the outgoing SEs always develops close to the surface. For Ω_Diel_ < 0 (negative charging of the dielectric), the potential wells appear over the conductor domains, as seen in the top panel of Fig. 4. However, if Ω_Diel_ > 0 (positive charging of the dielectric), the potential wells are located over the dielectric domains. In both cases, the potential wells are separated by a distance of 2 W and the potential approaches zero as the distance from the surface increases; see the bottom panel of Fig. 4. As stated above, the value of the electric potential depends only on W and $\overline{{V}_{S}}$. For example, the potential at a vertical distance of 10 W will always be one-fourth of $\overline{{V}_{S}}$. This also means that the electric potential at a certain distance from the surface for a specific Ω_Diel_ can be controlled by adjusting the domain size of the coating.

For any value of $\overline{{V}_{S}}$, the potential at the surface of the dielectric domains will peak at $2\overline{{V}_{S}}$, due to its almost linear growth along the surface of the dielectric. This relationship is shown in the inset of the bottom panel of Fig. 4. As stated above, we use a nearest-neighbour approximation to compute the electric field generated by the dielectric and conductor domains. The effect of this approximation is that the equipotential surfaces differ slightly from the conductor surface, see inset at the bottom panel in Fig. 4.

The landing energy of electrons incident on the dielectric domains is the difference between the energy generated by the e-gun and $e\overline{{V}_{S}}$. As we consider conductor domains to be grounded, the energy of incident electrons on the conductor domains is simply the e-gun energy. The trajectories of the primary electrons can also be modified by the surface charge of the sample. As can be seen in Fig. 4 (top and bottom panels), when the surface voltage is negative (notice that the ${\rm{V}}/\overline{{V}_{S}}$ ratio is always positive), potential barriers and potential wells develop over the dielectric and conductor domains respectively. If the primary electron energy is lower than the energy barrier on the dielectric domains, all primary electrons impact on the conductor domains.

The positions where SEs are generated are homogeneously distributed over the surface of the irradiated domains. The outgoing directions are generated following the generally assumed angular distribution of SEs (the Lambert cosine distribution)^26^. The energy spectrum of the SEs is given by

5 $$\rho (E)={\rho }_{0}\frac{E}{{(E+\varphi )}^{4}},$$

where ρ_0_ is a normalization factor and *ϕ* is the work function/affinity of the metal/dielectric domains^27^. For simplicity, we used *ϕ* = 2 eV and 4 eV for both the conductor and the dielectric domains, which means that the SE energy distribution peaks at ~1-5 eV.

After each pulse of PEs, the surface charge density accumulated on the dielectric domains changes according to

6 $${\Delta \Omega }_{Diel}=[({\sigma }_{Diel}-1)-{\sigma }_{Diel}\cdot {\xi }_{DD}-{\sigma }_{Cond}\cdot {\xi }_{DC}]\cdot {\Omega }_{Pulse}.$$

Ω_Pulse_ is the charge density of the incident pulse, and *σ*_Diel_ and *σ*_Cond_ are the uncharged secondary emission yields of the dielectric and conductor domains for a given primary energy. ξ_DD_ (ξ_DC_) is the fraction of secondary electrons that hit dielectric domains after being emitted by dielectric (conductor) domains. Similarly, ξ_CD_ (ξ_CC_) is the fraction of SEs that hit conductor domains after emerging from dielectric (conductor) domains. Finally, ξ_D_ (ξ_C_) is the fraction of secondary electrons that escaped the sample from dielectric (conductor) domains. The time constant, *τ*, was used to compute the charge remaining in the dielectric domains between consecutive pulses.

**Figure 5:**
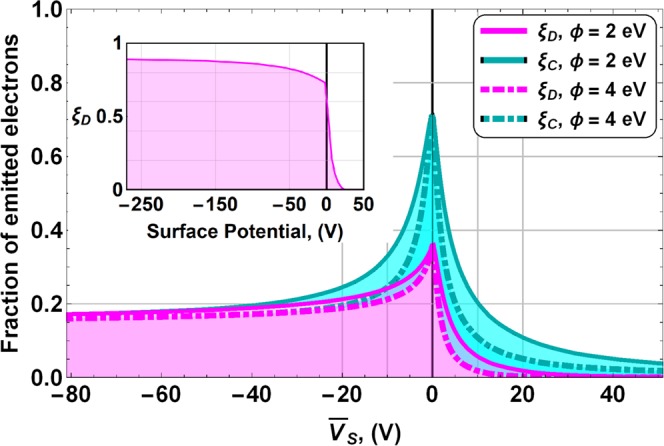
Fraction of emitted secondary electrons from dielectric domains ξ_D_ (magenta) and conductive domains ξ_C_ (cyan) as a function of the mean surface potential for two different work functions *ϕ* = 2, 4 eV. We can see that for both dielectric and conducting domains, the width of the peak increases with the work-function/affinity value, but the overall shape of the curve is the same. Inset: fraction of emitted electrons in a pure dielectric material as a function of the surface potential.

The total SEY of the sample is computed as the average of ξ_D_ and ξ_C_ multiplied by the corresponding uncharged *σ*_Diel_ and *σ*_Cond_ at a given primary energy. ξ_D_ and ξ_C_ are described in Fig. 5 as a function of $\overline{{V}_{S}}$. We can see that both parameters exhibit a peak when the sample is uncharged. The reason why it does not rise to one is that the roughness of the sample inhibits a part of the SE emission. At positive surface potentials, $\overline{{V}_{S}} > 0$, the emission of SEs from the composite coating can be inhibited. This is also the case for a pure dielectric surface, as shown in the inset of Fig. 5, where SEs are captured when positive charge accumulates in the surface. When $\overline{{V}_{S}} < 0$, SEs from conductor domains are effectively trapped, but some SEs from dielectric domains can still be emitted. This is very different from the pure dielectric case, where all SEs are emitted if the surface has negative charge. For comparison, results for work functions (affinity in the case of dielectrics) *ϕ* = 2 eV and 4 eV are shown in Fig. 5. We can appreciate that the width of the peak located at $\overline{{V}_{S}}=0\,V$ increases with the work function/affinity, but the shape of the curve remains similar.

As can be observed in Fig. 5, the higher the positive surface voltage (or negative surface voltage), the better the coating efficiency at capturing SEs emitted by dielectric and conductive domains. Thus, by the time tertiary electrons could start to be important, more of them are deflected toward the sample due to the accumulated charge. Therefore, it is expected that tertiary electrons make a negligible contribution to the total emitted current.

**Figure 6:**
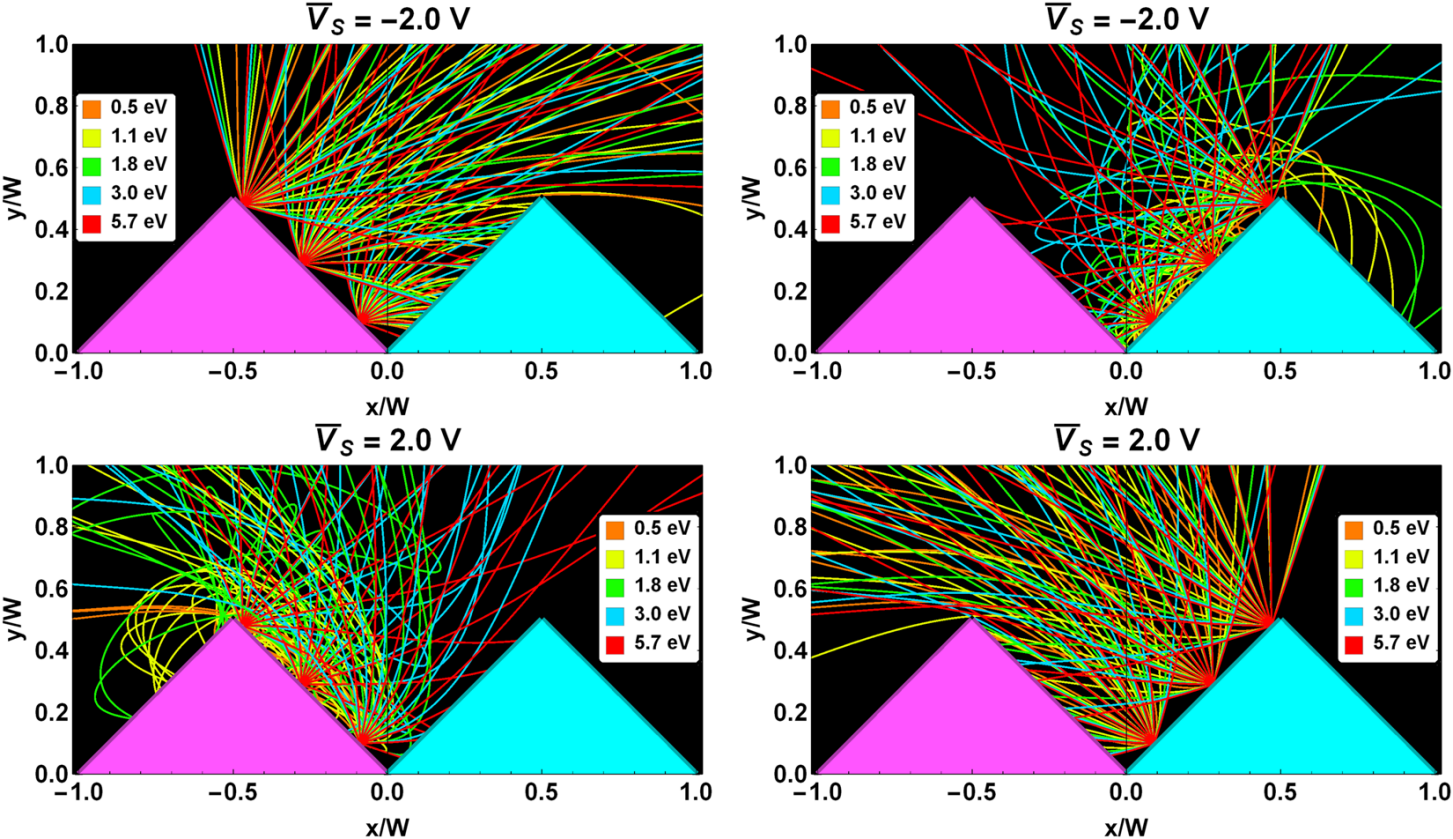
Potential well and electron trajectories. The spatial dimensions are normalized to the prism width, W (see text). Magenta (cyan) triangles represent dielectric (conductor) domains. Computed trajectories of secondary electrons emitted by the dielectric and conductor domains. For clarity, only three emission points are displayed. The top row shows trajectories for the negative surface potential, $\overline{{{\boldsymbol{V}}}_{{\boldsymbol{S}}}}=-2\,V$, and the bottom row trajectories for the positive surface potential, $\overline{{{\boldsymbol{V}}}_{{\boldsymbol{S}}}}=2\,V$. The energies of the SEs (from less than 1 eV to 6 eV) are given by the trajectory colours.

In Fig. 6 we can observe computed trajectories of the secondary electrons emitted from both the conductor and dielectric domains for $\overline{{V}_{S}}=-2\,V$ and $\overline{{V}_{S}}=+2\,V$. In the case of $\overline{{V}_{S}} < 0$, the SEs emitted from the dielectric domains (top left panel) follow nearly linear trajectories close to the top of these domains. Due to the surface roughness, some SEs from the dielectric domains impact the conductor domains. Noticeably, for $\overline{{V}_{S}} < 0$, an important fraction of SEs from the conductor domains (top right panel) return to the conducting surface. This is due to the electric field that arises in the grooves of the coating between neighbouring charged dielectric and grounded conductor domains. The radius of curvature of the outgoing SE trajectories increases with their energy, and so does their escape probability.

In Fig. 6, we can also observe that the trajectories of SEs emitted from the dielectric at $\overline{{V}_{S}} < 0$ are similar to those emitted from the conductor at $\overline{{V}_{S}} > 0$, and vice versa.

## Discussion

We perform simulations of the SEY curves for *Samples 1*, *2* and 3, using both the continuous and pulsed methods. The simulation parameters are the SEYs of the uncharged dielectric and conductor domains *σ*_Diel_ and *σ*_Cond_, the time constant τ of the discharging process, the size W of both dielectric and metals domains, the primary electron flux, and the total dose.

As described in SEY Results section, the discharging process has a time constant of ~100 s. We use a domain size of 1 mm for *Sample 3*, and 1 μm for *Sample* 1 and *Sample 2*. These values correspond approximately to the diameters of the dielectric domains seen in SEM images (Fig. 1). The incident electron flux for the continuous method is 2 nA/cm^2^ for all three samples, and the total dose is 10 nC/mm^2^ for all simulations. A second measurement and simulation with total dose of 100 nC/mm^2^ was also performed in *Sample* 1. The SEY values of the uncharged samples and constituents are shown in Table 1. The results of the simulations based on this geometric model (red) are very similar to the experimentally measured SEY curves (blue), as seen in Fig. 2.

The model correctly predicts the drop in SEY at low primary energies in the three samples. As SEY < 1 at primary energies lower than E_1_, the dielectric domains charge negatively. As can be seen in Figs 5 and 6 negative surface potentials reduce considerably the emitted secondary electrons explaining the aforementioned drop in the SEY.

This model explains why *E*_1_^*C*^ shifted towards higher energies when we used a higher incident dose in *Sample 1*, Fig. 2. The higher the dose, the more negative charge builds up in the dielectric domains; this slows down the PEs, so the incident energy stays in the *σ*_Diel_ < 1 domain longer. A larger surface charge also creates deeper, more effective potential wells, allowing the material to achieve lower SEY values. The degree to which *E*_*1*_^*C*^ differs from *E*_*1*_ depends on this interplay between the incident flux, the total dose, the different SEY values of the domains, and the conductivity of the dielectric material

The extremely low yields measured in *Sample 3*, even at high primary energies, can be also reproduced by our model (see Fig. 2). As discussed in relation to Eq. (4), for a similar incident electron flux higher voltages can be obtained if the domain size is larger as in *Sample 3* (about 1 mm). In this case, $e\overline{{V}_{S}}$ is able to follow closely the PE energy until the end of the measurement. Thus, its dielectric domains are always charging negatively, so the SEs are efficiently trapped by potential wells and dissipated through the conductor domains.

Dielectric breakdown is not considered in this model. Breakdown is not expected for electric fields weaker than 10 MV/m^28^. In the simulations, the maximum voltages experienced by *Sample 1* and *Sample* 2 are ~10 V; a particle size of ~1 µm gives a field strength of ~10 MV/m, close to but still below the typical dielectric breakdown limit. *Sample* 3 experienced even lower electric fields, ~1 MV/m, (potentials up to 1000 V, particle size ~1 mm).

## Conclusions

We achieved extremely low SEY values for rough metal/dielectric coatings, *σ* = 0.2, even for primary electron energies near 1 keV. We measured SEY curves for three very different composite coatings with well-differentiated dielectric and conductor domains.

We proposed a unique model of secondary electron emission that explains physical experiments and give us insight into the extremely low SEY values obtained in the experiment for metal/dielectric composite coatings. The model is based on both surface roughness and the electric field that arises between charged dielectric and grounded conductor domains of the composite coating. The dynamic SEY behaviour of these coatings depends on the size of the domains, the time constant of the discharging process, the incident electron flux, and the SEY of the uncharged constituents of the composite.

One of the main practical outcomes of the model is that it explains that an important decrease in the SEY of the metal/dielectric coatings can be achieved by only a small voltage difference (<2 V) between the dielectric and conductor domains. This behaviour, along with the observed quick decay of the generated external potential, paves the way to low-SEY composite coatings with conducting properties suitable for many critical technological applications.

## Supplementary information

SEY Dataset

## Data Availability

All experimental SEY data generated during this study is included in this published article (and its Supplementary Information files).
